# Is There a Unified Etiology of Hypoplastic Left Heart Syndrome? Evaluating Genetic, Structural, and Hemodynamic Models of Disease Initiation

**DOI:** 10.3390/pathophysiology33020033

**Published:** 2026-05-20

**Authors:** Reese Leonhard, Zachary Beau Phillips, Jamie Wilson, Zaid Abu-Mowis, John DiGiorgi, Epiphany N. Wilson, Zane Borenstein, Laura Wilson, Richard Tang, Elizabeth H. Stephens, Adrian Crucean, Michael S. Shillingford, Giles J. Peek, Mark Steven Bleiweis, J. Steven Alexander, Jeffrey Phillip Jacobs

**Affiliations:** 1 Congenital Heart Center, Departments of Surgery and Pediatrics, UF Health Shands Hospital, University of Florida, 1600 SW Archer Rd, Gainesville, FL 32608, USA; reeseleonhard@gmail.com (R.L.); zachary.phillips@lsuhs.edu (Z.B.P.); jwilson5@ufl.edu (J.W.); zabumowis@ufl.edu (Z.A.-M.); digiorgi.j@northeastern.edu (J.D.); epiphanyw129@gmail.com (E.N.W.); or zborenstein71@gmail.com (Z.B.); wilsonl@ufl.edu (L.W.); m.shillingford@ufl.edu (M.S.S.); gilespeek@ufl.edu (G.J.P.); bleiweis@ufl.edu (M.S.B.); 2 Louisiana State University Health Sciences Center at Shreveport, School of Medicine, 1501 Kings Highway, Shreveport, LA 71103, USA; jonathan.alexander@lsuhs.edu; 3 Pediatric Cardiac Surgery, Children’s Hospital of Pittsburgh, University of Pittsburgh Medical Center, Pittsburgh, PA 15213, USA; tangrc@upmc.edu; 4 Department of Cardiovascular Surgery, Mayo Clinic, Rochester, MN 55905, USA; stephens.elizabeth@mayo.edu; 5 Department of Paediatric Cardiac Surgery, Birmingham Women’s and Children’s Hospital, Birmingham B4 6N4, UK; adrian.crucean@nhs.net

**Keywords:** hypoplastic left heart syndrome, HLHS, congenital heart disease, cardiac development, hemodynamics, genetic mutation, endocardial fibroelastosis, valvar malformation, myocardial dysplasia, cardiac progenitor cells, fetal cardiology

## Abstract

**Background**: Hypoplastic left heart syndrome (HLHS) is defined as “a spectrum of congenital cardiovascular malformations with normally aligned great arteries without a common atrioventricular junction, characterized by underdevelopment of the left heart with significant hypoplasia of the left ventricle including atresia, stenosis, or hypoplasia of the aortic or mitral valve, or both valves, and hypoplasia of the ascending aorta and aortic arch”. Without treatment, HLHS is usually lethal in the neonate. Many hypotheses have been advanced to explain the etiology of HLHS; however, no single theory appears to fully explain the phenotypic variability seen in HLHS. Furthermore, many of these theories offer no explanations regarding the ***precipitating events*** which lead to the development of HLHS. **Objective**: This review considers and critically evaluates the strengths and weaknesses of the leading theories proposed to explain the pathogenesis of HLHS—including ***hemodynamic disturbances***, ***primary myocardial structural defects***, ***valvar malformations***, and ***genetic or epigenetic alterations that may provoke developmental and anatomic abnormalities***. After presenting each model, we propose a novel, comprehensive, and data-driven framework which may assist researchers in developing models for the pathogenesis of the various subtypes of HLHS. **Methods**: Key findings from human fetal imaging, histopathology, genetic studies, and animal models were considered, as well as the hypothetical contribution of each in observed HLHS phenotypes. The rationales for these findings as causal factors initiating individual HLHS patterns, as well as how they might contribute to HLHS in general, were critically analyzed. **Results**: The ***flow theory*** is strongly supported by animal models and in utero interventions that demonstrate the impact of altered hemodynamics on cardiac morphogenesis. However, the flow theory fails to identify initial causes of disturbed flow or related histological features of HLHS like endocardial fibroelastosis. The ***myocardial and valve-first models*** suggest an important role in developmental defects, but do not necessarily have a strong experimental basis that provides explanations for how they mediate HLHS. ***Genetic studies*** in patients with HLHS have identified several candidate causal mutations. However, such genetic causes of HLHS exhibit incomplete phenotypic penetrance and clinical impact. A ***multifactorial framework*** attempts to integrate these diverse mechanisms and may provide the most coherent explanation that can accommodate the heterogeneity and variable presentation of HLHS. Such a framework may identify multiple forces that drive disease but does not provide useful pathways for future research about HLHS. **Conclusions**: No single hypothesis has fully explained how HLHS is initiated, progresses, and presents with the clinical conditions that are encountered by cardiac surgeons and cardiologists. The most current models suggest that the spectrum of HLHS reflects a***complex interaction between genetic susceptibility, flow-dependent cardiac remodeling, and environmental factors in utero***. ***A multifactorial model integrates these diverse mechanisms and may provide the most coherent explanation for the various phenotypic variations in HLHS.*** Based on our analysis of the most current data and the strengths and weaknesses of the current theoretical frameworks, we propose a novel research strategy aimed at identifying specific cardiac progenitor cell populations whose dysregulation may represent a unifying explanation for the etiology of the various phenotypes of HLHS. Based on the arguments made throughout this manuscript that evaluate the various genetic, structural, and hemodynamic models of initiation of disease, we believe that the ***significant phenotypic variability across the spectrum of HLHS (i.e., the different anatomic subtypes for “classic” HLHS) most likely reflects different underlying etiologies and mechanisms***. At the very least, it is very likely that the ***timing*** of the insult is critical in determining anatomic subtype. Based on the published data and the arguments within this manuscript, it seems ***naive to think that there is a single unifying mechanism explain all forms of HLHLS***.

## 1. Introduction

The ***International Paediatric and Congenital Cardiac Code*** (***IPCCC***) and the Eleventh Revision of the ***International Classification of Diseases*** (**ICD-*11***) provide the following definition of Hypoplastic Left Heart Syndrome (HLHS) [1,2,3,4,5,6,7,8]:

“A spectrum of congenital cardiovascular malformations with normally aligned great arteries without a common atrioventricular junction, characterized by underdevelopment of the left heart with significant hypoplasia of the left ventricle including atresia, stenosis, or hypoplasia of the aortic or mitral valve, or both valves, and hypoplasia of the ascending aorta and aortic arch.”

The most critical aspect of this definition of HLHS is that it recognizes HLHS as a spectrum of malformations that is not defined by one distinct morphology. Indeed, the umbrella term “HLHS” encompasses several different cardiac phenotypes and associated anatomic arrangements, and each of these anatomic arrangements may have different etiologies and different outcomes.

HLHS may be subclassified into the following 5 subtypes [5,9]:

- Hypoplastic left heart syndrome (HLHS), Aortic atresia + Mitral atresia (MA/AA)
- Hypoplastic left heart syndrome (HLHS), Aortic atresia + Mitral stenosis (MS/AA)
- Hypoplastic left heart syndrome (HLHS), Aortic stenosis + Mitral atresia (MA/AS)
- Hypoplastic left heart syndrome (HLHS), Aortic stenosis + Mitral stenosis (MS/AS)
- Hypoplastic left heart syndrome (HLHS), Without intrinsic valvar stenosis or atresia (Hypoplastic aortic valve + mitral valve + left ventricle) = Hypoplastic left heart complex = HLHC

Although these subtypes of HLHS are mostly defined by the morphology of their valves, the anatomy of the left ventricle is equally important and changes drastically depending upon the subtype in question. For example, the left ventricular morphology that is associated with the MA/AA subtype of HLHS is characterized by a hypoplastic “slit-like” left ventricle, whereas the left ventricular morphology that is associated with the MS/AA subtype of HLHS is characterized by an underdeveloped left ventricle with hypertrophy [9]. Under the microscope, these anatomic differences can further be differentiated by the presence or absence of endocardial fibroelastosis, a rare cardiac abnormality in which the endocardium is thickened due to excessive fibrous and elastic tissue; endocardial fibroelastosis is seen in certain subtypes of HLHS and will be discussed in detail later in this review. The importance of these distinctions cannot be understated. As our subsequent discussion of the leading theories of the pathogenesis of HLHS progresses, it will become evident that each major hypothesis about the etiology of HLHS can describe certain subtypes of HLHS with clarity and plausibility but ultimately fails to account for other subtypes of HLHS accurately.

Treatment of HLHS represents one of the great ‘success stories’ in medicine and surgery. HLHS was once a uniformly fatal disease, but HLHS now has reported survival at one year of age of over 90% in some centers [10,11,12], although tremendous variation in strategies of treatment and outcomes still exists [13,14,15,16,17]. Data is lacking about rates of survival into adulthood for patients with HLHS; however, a recent analysis of linked data from The Society of Thoracic Surgeons Congenital Heart Surgery Database and data from the National Death Index was presented at 2026 annual meeting of The Society of Thoracic Surgeons on Thursday, 29 January 2026 and reported an overall median survival of 26.8 years in an analysis of 16,728 patients with HLHS.

**Figure 1:**
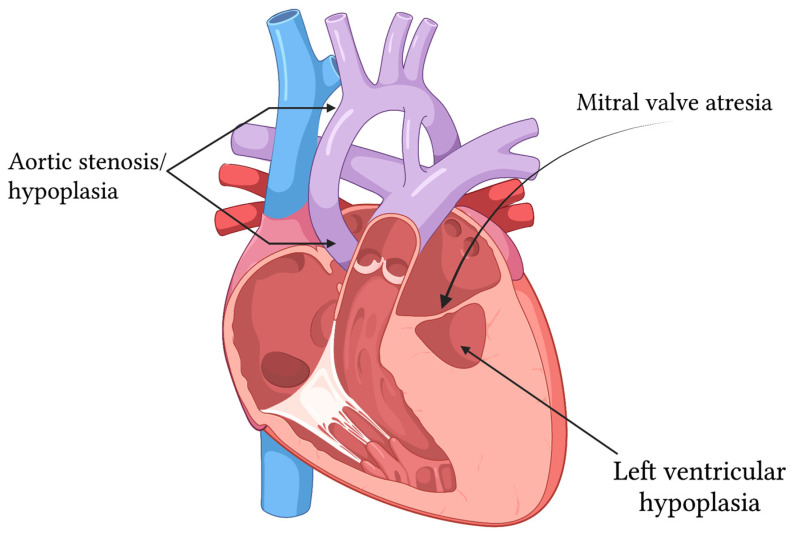
Cartoon depiction of hypoplastic left heart syndrome (HLHS) prior to any surgical intervention. Schematic illustration of a heart with HLHS demonstrating a diminutive left ventricle, hypoplastic ascending aorta, and atresia or severe stenosis of the mitral and aortic valves. The drawing emphasizes the underdeveloped left-sided cardiac structures that define the syndrome, while the right ventricle and pulmonary artery are proportionally enlarged to reflect their role in supporting systemic circulation through ductal flow. (In this drawing of a heart with HLHS prior to any surgical intervention, the blue coloring of the superior caval vein [i.e., superior vena cava] and inferior caval vein [i.e., inferior vena cava] represents deoxygenated blood returning from the systemic circulation to the right atrium; the red coloring of the pulmonary veins represents oxygenated blood returning from the lungs to the left atrium, and the purple coloring of the Pulmonary Artery, Aorta, and Arterial Duct [i.e., Ductus Arteriosus] represent mixing of venous and arterial blood [i.e., mixing of deoxygenated and oxygenated blood]). Created in BioRender. Leonhard, R. (2025) https://app.biorender.com/illustrations/68b04841ff303c5da4be0c9d.

**Figure 2:**
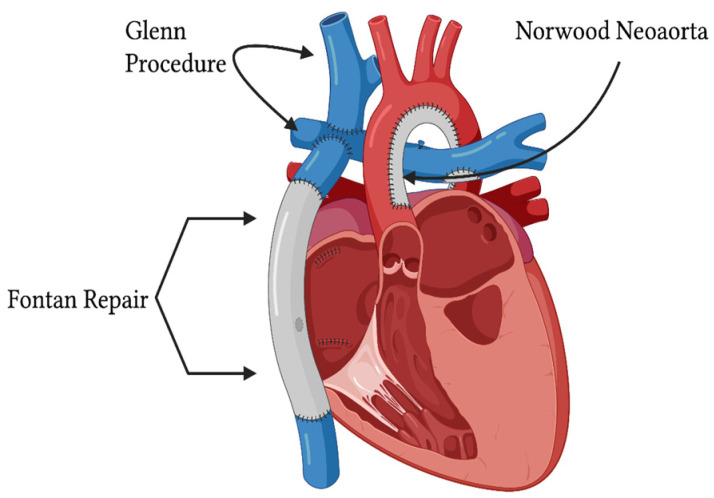
Cartoon depiction of staged palliation for hypoplastic left heart syndrome (HLHS) shown on a single heart. The diagram illustrates the characteristic HLHS anatomy overlaid with the three sequential stages of palliation. Stage I (Norwood procedure) is represented by reconstruction of the diminutive aorta with the pulmonary artery to form a neoaorta and placement of a systemic-to-pulmonary shunt. Stage II (Bidirectional Glenn) is depicted with the superior vena cava connected directly to the pulmonary arteries, reducing right ventricular volume load. Stage III (Fontan completion) is shown with the inferior vena cava routed to the pulmonary arteries, completing total cavopulmonary connection. Collectively, the figure highlights the progressive surgical reconfiguration that enables systemic output through the single right ventricle while establishing passive pulmonary venous return. (In this drawing of a heart with HLHS after surgical intervention with a Norwood [Stage 1] Operation, a Glenn ([Stage 2] superior cavopulmonary anastomosis, and a completion Fontan [Stage 3} total cavopulmonary connection, the blue coloring of the superior caval vein [i.e., superior vena cava], inferior caval vein [i.e., inferior vena cava], and right and left branch pulmonary arteries represents deoxygenated blood returning from the systemic circulation to the pulmonary arteries; the red coloring of the pulmonary veins and neoaorta represents oxygenated blood returning from the lungs via the pulmonary veins to the left atrium and then being pumped to the body via the aorta, and the grey coloring the bioprosthetic patch material of the Fontan tube, neoaortic patch, and main pulmonary arterial patch). Created in BioRender. Leonhard, R. (2025) https://app.biorender.com/illustrations/687fe068ae2257171e69ec40.

Prior to the 1980s, HLHS was fatal. In 1983, the first successful surgical treatment of HLHS was reported in *The New England Journal of Medicine* by William I. Norwood in a manuscript titled “*Physiologic repair of aortic atresia-hypoplastic left heart syndrome*”; this groundbreaking operation is now known as the Norwood (Stage 1) Operation [18]. Following the Norwood (Stage 1) Operation (Figure 1), patients usually undergo two sequential stages of surgical palliation, known as the Glenn Operation and the Fontan Operation, respectively (Figure 2). In 1985, in *JAMA*, Leonard Bailey reported the first baboon-to-human cardiac xenotransplantation in a neonate with HLHS, which was performed on 26 October 1984 and set the stage for both neonatal cardiac transplantation in general and cardiac transplantation as a treatment option for HLHS [19]. In 2002, the hybrid approach for treating HLHS, using stenting of the arterial duct and banding of the pulmonary arteries, was reported in *Circulation* [20].

These three treatment strategies all serve a role in the management of neonates with HLHS, and have converted the prognosis of HLHS from a previously fatal disease to a currently treatable disease:

(1) staged palliation with Norwood (Stage 1) Operation, Glenn (Stage 2) Operation, and completion Fontan (Stage 3) Operation;
(2) cardiac transplantation; and
(3) the hybrid approach with banding of the branch pulmonary arteries ± ductal stenting

Still, between 1 January 2014–31 December 2017, inclusive, 2737 Norwood (Stage 1) Operations were performed at 105 hospitals participating in The Society of Thoracic Surgeons Congenital Heart Surgery Database (STS-CHSD), with an aggregate rate of Operative Mortality of 15.0% [21]. These outcomes have continued to improve, and the Spring 2023 Feedback Report of STS-CHSD analyzed data from 1 January 2019–31 December 2022, inclusive, and included 1753 Norwood (Stage 1) Operations performed, with an aggregate rate of Operative Mortality of 210/1753 = 11.98% [22].

Longitudinal aggregate outcomes following the treatment of HLHS are more challenging to obtain. The Single Ventricle Reconstruction Trial, funded by the National Institutes of Health of the United States of America, documented a one-year survival of 64% in patients undergoing Norwood (Stage 1) Operation with modified Blalock-Taussig-Thomas (BTT) systemic-to-pulmonary artery shunt and 74% in patients undergoing Norwood (Stage 1) Operation with Sano right ventricle to pulmonary artery conduit [23,24,25]. Outcomes of patients with HLHS have improved tremendously over the past several decades, and substantial research has supported these improvements [23,24,25,26,27,28,29,30,31,32,33,34,35,36,37,38,39,40,41,42,43,44,45,46,47,48,49,50,51,52,53,54,55,56,57,58,59,60].

An understanding of the etiology of HLHS, and specifically the etiology of the various subtypes of HLHS, as well as the genetic, structural, and hemodynamic models of the initiation of the subtypes of HLHS, can potentially lead to additional advances in the treatment and maybe the eventual prevention of this challenging disease. However, while HLHS has become more treatable through surgical interventions, it remains incurable. A major obstacle toward discovering curative therapies for HLHS is that the etiology of each HLHS subtype remains largely unknown. As of now, leading theories about the etiology and the initiation of the anatomic variants of HLHS only explain certain aspects of the pathogenesis of HLHS, failing to capture definable, developmentally traceable, mechanistic explanations for the etiology of each of the subtypes of HLHS. Without a complete mechanistic understanding of the molecular and developmental pathways involved in the specific subtypes of HLHS, earlier detection and curative therapeutic interventions are less likely. Consequently, this review summarizes the major theoretical frameworks that have been proposed to explain the etiology of HLHS and evaluates how the strengths and limitations of each theoretical framework might apply to the different subtypes. Building on these insights, we propose a new, hypothesis-driven framework for future research that integrates data-supported principles from existing theories. This approach suggests that the initiation and progression of each subtype of HLHS may be explained by definable pathogenic mechanisms understood at the cellular level.

## 2. Overview of the Embryonic Heart as We Know It

**Figure 3:**
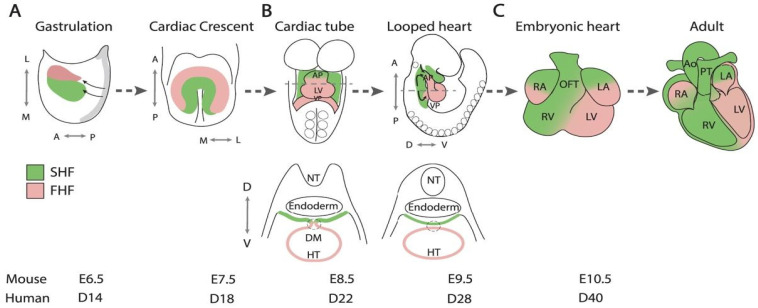
Schematic of cardiac morphogenesis highlighting contributions from the first (pink) and second (green) heart fields during mouse heart development. (**A**) Formation of the primitive heart tube from early progenitor cells. (**B**) D-looping and extension of the heart tube. (**C**) Expansion of cardiac chambers and formation of septal structures. Abbreviations: FHF, first heart field; SHF, second heart field; L, lateral; M, medial; A, anterior; P, posterior; D, dorsal; V, ventral; AP, arterial pole; VP, venous pole; HT, heart tube; RA, right atrium; LA, left atrium; RV, right ventricle; LV, left ventricle; NT, neural tube; DM, dorsal mesocardium; OFT, outflow tract; Ao, aorta; PT, pulmonary trunk; E, mouse embryonic day; D, human embryonic day. Reproduced from [73] Licensed under CC BY 4.0.

The search for such a mechanistic understanding of HLHS begins with a thorough understanding of normal cardiac development during early gestation. The embryonic heart first takes shape in the third week of gestation with the formation of the ***primitive streak***. The primitive streak is a structure composed of epiblast cells, a group of pluripotent stem cells that progenerate all subsequent layers of embryonic tissue. Through a process known as gastrulation, occurring at around day 16, the primitive streak differentiates from a single layer of epiblast cells into the ectoderm, mesoderm, and endoderm, which will give rise to various organ systems in the adult human. At this stage of development, the heart is derived entirely from mesodermal cells. More specifically, the heart is derived from cells from the splanchnic layer of the lateral plate mesoderm [61]. In the splanchnic layer of lateral plate mesoderm, the most critical population of cells for the development of the left heart is born, known as the First Heart Field (FHF). This population of cells is directly responsible for the development of Left Ventricular (LV) myocardium and acts as a scaffold for the anterior Second Heart Field (SHF), which contributes significantly to the formation of the left and right ventricular outflow tracts [62] (Figure 3). The FHF is acted on by a network of key transcription factors—including NKX2.5, MEF2, GATA, Tbx, and Hand—which guide the differentiation of pre-cardiac cells into chamber-specific cardiomyocytes, endocardial endothelium, and smooth muscle cells [62]. Importantly, mutations in these networks have been associated with development of congenital heart disease, including HLHS. Further detail about these gene networks will be discussed in a subsequent section of this review. As development proceeds, the linear heart tube forms and undergoes rightward looping (D-looping), establishing the left-right orientation necessary for proper chamber positioning.

At the time that primary looping occurs, the first evidence of the development of cardiac valves arises. At this point in development, the looped heart tube is composed of an inner layer of endocardial cells and an outer layer of myocardial cells. separated by an extracellular matrix (ECM) known as the cardiac jelly [63]. When primary looping begins, the myocardial cell layer at the primitive atrioventricular junction and the primitive outflow tract begin to downregulate their chamber-specific gene products and instead increase their production of ECM, causing the cardiac jelly to swell. These swellings form the first structures in the heart that prevent backflow of blood and are known as “endocardial cushions” [64]. The formation of these endocardial cushions is regulated by a process known as endocardial-to-mesenchymal transition (EMT). EMT is a process by which the endocardial cells within the cushions break their cell to cell connections and migrate into the cardiac jelly, simultaneously changing their cell properties to act like fibroblasts that will eventually mature into the fibrous tissue that comprises mature atrioventricular valves [65]. The exact mechanisms by which the post-EMT mesenchymal cells become the mature atrioventricular valvar tissue remain unclear. However, lineage tracing studies have demonstrated that the cells of the mature atrioventricular valves are derived mostly from this endocardial origin, with some contributions from the epicardium. Cells derived from cardiomyocytes do not contribute to the atrioventricular valves [66,67].

The developmental programs of the atrioventricular valves and semilunar valves are broadly similar, yet these valvar developmental programs diverge in several critical steps. After endocardial-to-mesenchymal transition, mesenchymal progenitors destined to form both valve types migrate into the cardiac jelly and establish the cell polarity needed for proper valvar morphology, a process directed by genetic patterning cues, chemoattractant gradients, and biomechanical forces [68]. Although this invasion–remodeling sequence is shared between the atrioventricular valves and the semilunar valves, the major distinction lies in the cellular lineages that contribute to each structure. Atrioventricular valves arise entirely from endocardial cushion mesenchyme, whereas semilunar valves incorporate an additional, essential population of cardiac neural crest cells. These neural crest–derived cells populate the outflow tract cushions and are indispensable for proper formation of the semilunar leaflets and septation of the outflow tract [66,69].

While the valves are undergoing maturation, the primitive ventricles proceed through an important process called trabeculation [70]. Trabeculae are a network of myocardial cells, covered by an endocardial layer, that project into the ventricular lumen and increase the ability of the heart to generate cardiac output [71]. The development of trabeculae occurs in two stages. The first, known simply as trabeculation, occurs as the myocardial/endocardial cell clusters expand toward the luminal side of the ventricle to form distinct, primitive trabeculae. Next, these primitive trabeculae undergo a remodeling process by which their growth is directed radially toward the cardiac wall, rather than towards the luminal side of the ventricle [70,72]. Similarly to the development of the heart valves, the process of trabeculation fails without a proper endocardium, and in studies of zebrafish with inhibited endocardial cell lineages, trabeculation ceased and the developing ventricles became malformed [72].

## 3. Flow/Hemodynamic Theory of Pathogenesis

Cardiac development is not solely dependent on genetic signaling pathways. The embryonic cardiac cells take cues from the environment, and one of the most important of these environmental cues is hemodynamic forces. Because of the importance of hemodynamic forces to the normal growth and morphogenesis of the heart, the ***hemodynamic (or flow) theory*** for the development of HLHS has been one of the most longstanding and experimentally supported models for disease initiation. In this section, we review foundational and recent literature supporting the hemodynamic theory, commonly referred to as the “no flow, no grow” theory, and we focus on animal models, experimental interventions, and their relevance to the pathogenesis of HLHS in humans.

### 3.1. Mechanistic Appeal and Clinical Correlation

**Figure 4:**
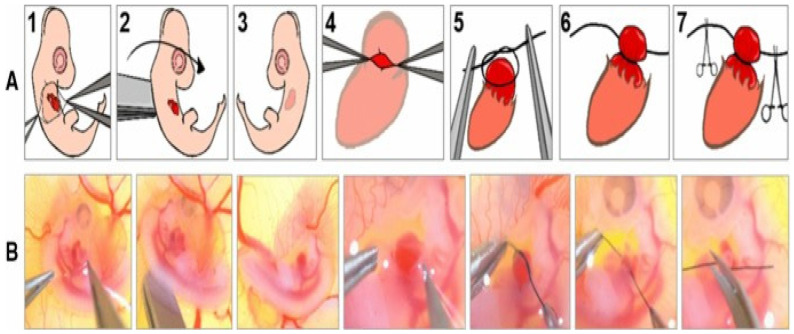
Image demonstrating the methodology of left atrial ligation (LAL) in avian embryonic cardiac models. (**A**) A cartoon representation of LAL procedure, (**B**) Photographs of embryonic chick embryo undergoing LAL procedure. Reproduced from [80]. Licensed under CC BY 4.0.

**Figure 5:**
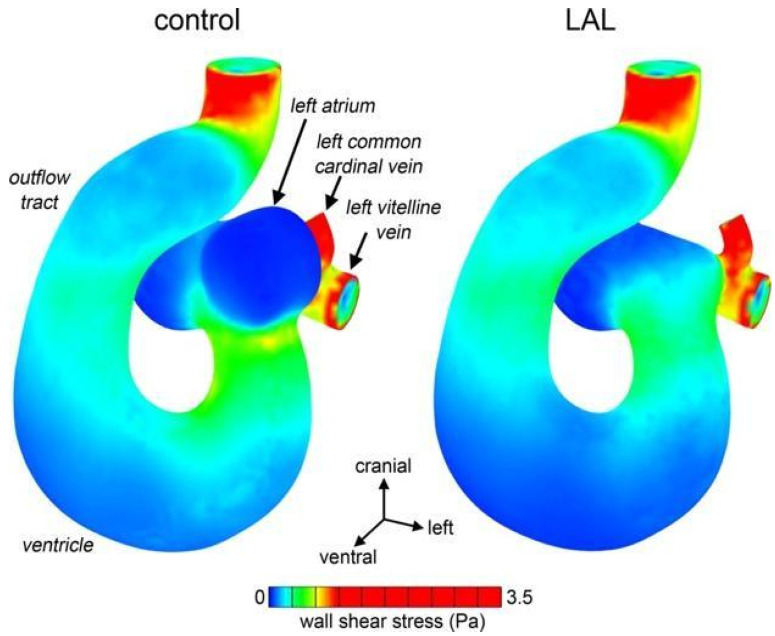
Wall shear stress (WSS) distributions in normal versus left atrial ligated (LAL) chick embryos. LAL significantly alters the hemodynamic environment by reducing WSS in left-sided cardiac structures and increasing localized oscillations, which are hypothesized to contribute to hypoplastic remodeling. Adapted from [82]. Licensed under Creative Commons Attribution 4.0 (CC BY 4.0).

Literature that supports this hemodynamic (or flow) theory extends back to the 1960s and 1970s, with descriptions of two techniques that experimentally altered blood flow in chick embryos. Harh and colleagues demonstrated that obstructing flow in the left atrioventricular canal of chick embryos induced left heart hypoplastic morphology [74]. Further, Rychter and colleagues used a technique that has since evolved into left atrial ligation (LAL) in chick embryos to similarly impact the development of left heart structures, including the left ventricle and the mitral valve, and also to impact normal aortic flow [75]. LAL has become a robust and reproducible experimental method for inducing HLHS-like phenotypes in several different species. By mechanically obstructing left atrial inflow, LAL disrupts normal pressure gradients and flow patterns that are essential for the development of cardiac chambers and valves. This intervention is typically done late in development, and across numerous studies, this intervention has consistently led to underdevelopment of the left ventricle, mitral valve, and ascending aorta, mirroring the clinical phenotype of HLHS [76,77,78,79,80,81]. Beyond gross morphology, LAL models (Figure 4 and Figure 5) have revealed flow-dependent cellular and molecular responses, highlighting mechano-transduction as a crucial regulator of cardiac patterning during development [76].

Recent work by Salman and colleagues reinforces this causative link. In embryos subjected to LAL, Salman and colleagues documented significant reductions in left ventricular mass, atrioventricular valvar dimensions, and average atrioventricular canal velocity.

Since LAL is performed later in development, the observed disruptions to the left-sided structures are much more attributable to mechanical alterations rather than genetic mutations and can be considered the primary driving force in the development of certain subtypes of HLHS [80]. Moreover, recent work by Rahman and colleagues using a mouse model of HLHS provides strong supportive data for the hemodynamic theory [81]. Their iteration of LAL, which induces disruption of normal flow via thrombus injection, consistently produces underdevelopment of the left ventricle in conjunction with hypoplasia of the aortic valve and aortic arch. This iteration distinguished itself from surgical LAL by producing aortic hypoplasia, which is a characteristic of HLHS that was not reproduced by the initial methods. The authors concluded from these studies that the hemodynamic theory could explain subtypes of HLHS that were characterized by aortic malformations that extended beyond just observable flow disruptions like those produced in the original form of LAL [81].

Finally, the clinical translation of flow theory provides some of its most compelling support. In the early 2000s, in utero balloon aortic valvotomy for fetuses with critical aortic stenosis demonstrated that restoring forward flow through the left heart could promote growth of left-sided structures, resulting in postnatal biventricular circulation in select cases [83]. This intervention, initially performed in a small number of high-risk fetuses, has since been refined and adopted as a viable strategy to prevent progression to HLHS in select fetuses at risk for HLHS. A recent review by Guseh and Tworetzky highlights how improvements in patient selection, procedural success, and long-term outcomes have transformed this technique into a meaningful therapeutic option for modifying HLHS trajectory in select fetuses in utero [84].

The success of fetal cardiac intervention—predicated entirely on restoring normal hemodynamics—provides direct clinical validation of the flow theory. The data demonstrate that disturbed flow is a modifiable driver of disease progression, independent of genetic predisposition. These experimental approaches suggest that hemodynamic forces and structural changes can reciprocally interact during development to drive HLHS phenotypes. However, it is important to understand that only a select subset of patients responded positively to in utero balloon aortic valvotomy and subsequently developed biventricular circulation. It is not known whether the failure of some patients to respond to in utero balloon aortic valvotomy is because

- the timing of the intervention is currently only possible at 20 weeks or later, or
- restoring normal prograde flow into the LV cannot overcome other factors at play in the progression of their disease process.

### 3.2. Flow/Hemodynamic Theory of Pathogenesis—Theory Limitations (Directionality and Developmental Blind Spots)

A major conceptual limitation for this hypothesis is directionality—that is, whether altered hemodynamics cause malformation of left-sided cardiac structures, or whether intrinsic structural or genetic abnormalities produce abnormal flow. For example, contributions of early valvar dysplasia or developmental defects in the myocardium that precede and provoke flow disturbances, rather than result from them, are consistent with heterogeneous contributions of structure, function, and flow, with many but not all fetuses in HLHS exhibiting measurable flow disturbances by the time they are diagnosed with HLHS. It is not uncommon that individuals with certain subtypes of HLHS, such as the MS/AA and MS/AS, will have normal standard first-trimester imaging on four-chambered heart views or nuchal translucency screenings, and develop features of HLHS in the late first to mid-second trimester of pregnancy [85,86]. Therefore, if flow disturbances were the initiating insult in all subtypes of HLHS, it might logically follow that hemodynamic changes would be observed in these early screening tests, rather than result from an already existing pathological anatomy.

It is possible that the hemodynamic changes are present at a critical developmental time-period, such as week 5 or week 6, and the pre-natal screenings are conducted too late to observe these early insults. Then. by the time the fetal heart is first seen, the changes induced by altered hemodynamic signaling have already exerted their initial effects and the pathogenic results are already developing, just not in a measurable way.

Conversely, the data from LAL experiments point toward altered hemodynamic signaling exerting its effects on the developing heart later in development, likely late enough to be observed on fetal screening. However, the data from LAL experiments, which might have resolved how these late features of the MS/AA subtype and MS/AS subtype develop, fail to provide a compelling answer. Observed underdevelopment of the mitral valve and aorta seen in the LAL studies mirrors human HLHS, but the decrease in LV volume appears to have an entirely different cause. In LAL models, underdevelopment of the LV is consistently attributed to myocardial thinning. However, in human HLHS, especially in the MS/AA and MS/AS subtypes, the LV volume decrease can be attributed to an increase in the myocardium [87]. It is important to note that in these subtypes, it is not currently known whether the increased myocardial growth is a result of hypertrophy, hyperplasia, or a combination of hypertrophy and hyperplasia. Still, such a vast difference in the makeup of the LV implies that the “second and third trimester HLHS” (MS/AA and MS/AS) are unlikely to be explained solely by the late-occurring hemodynamic disruptions like the hemodynamic disruptions induced in LAL models. One possible explanation for this observed discrepancy is a ‘two-hit’ model of disease pathogenesis. In this ‘two-hit’ framework, an initial genetic or environmental insult renders the developing fetal heart susceptible to the late subtypes of HLHS, and a subsequent disturbance in hemodynamic signaling later in gestation may then drive progression toward the MS/AA or MS/AS subtypes. However, this hypothesis remains speculative and has not yet been definitively established. Indeed, it is important to consider the concept that some of the phenotypes observed in HLHS are a developmental sequence (i.e., are secondary to earlier abnormalities upstream) as an alternative to the idea that each of the features arise independently as primary defects.

The flow theory also fails to fully explain histologic changes observed in the tissue in the MS/AA and MS/AS subtypes, including endocardial fibroelastosis (EFE) and myocardial disarray. Strong evidence points to a genetic etiology of these pathohistological changes. More specifically, mutations in the sarcomeric genes MYH7 and MYBPC3 have been heavily linked as the causal events for myocardial disarray and EFE in other neonatal cardiomyopathies related to HLHS [88]. Such findings imply that intrinsic myocardial and endocardial dysfunction may play a similar role in the pathogenesis of these late subtypes of HLHS that is independent of abnormal flow, which further complicates the hemodynamic explanation for the etiology of the MS/AA and MS/AS subtypes of HLHS. However during development, the endocardium signals to the underlying tissues to initiate growth and other processes. It is therefore possible that disrupted flow, detected by the endocardium, has effects on the subendocardial region and the myocardium. One such signaling pathway that has been well described in the literature is the NOTCH1 pathway. NOTCH1 acts as a mechanosensory gene network in the developing endocardium [89]. This endocardial-derived mechano-sensitive network is critical for normal ventricular trabeculation, valvar development, and outflow tract remodeling [90,91]. Interestingly, mutations in the NOTCH1 pathway have been associated with the development of HLHS, which will be discussed in a subsequent section of this review, although the exact mechanism of the relationship between the two has yet to be fully elucidated.

Another unanswered question in the framework of the hemodynamic theory is that the source of the disruption to normal hemodynamic signaling in the first place is not known. For example, many of the landmark studies used to formulate this theory of pathogenesis utilize surgically induced (or microembolization induced) LAL chick embryos. These models can effectively reproduce HLHS-like features, but since they artificially disrupt normal hemodynamics, they do not explain the etiologic insult in utero that would cause the downstream effects observed. In other words, these studies cannot explain what natural force acts as the “ligation event” to a developing fetal heart.

One such event may be a disruption of the placental–cardiac axis. This term is used to describe the interconnected signaling between the developing heart and the placenta. Current data suggests that disruptions in normal hemodynamics within the embryonic heart can disrupt normal vascularization of the placenta and vice versa, including hypoplasia of left-sided heart structures [92].

Finally, the success of in utero balloon aortic valvulotomy is not universally observed. Certain patients do not respond to this intervention and will go on to develop HLHS. This failure of many fetuses to respond to in utero balloon aortic valvulotomy might mean that by the time the in utero balloon aortic valvulotomy is performed, the damage is done and cannot be repaired by restoring flow at this late stage. Such results can imply that for a subset of patients, hemodynamic signaling may play a small or even insignificant role in the progression of disease. Although it is possible that an early disruption to normal hemodynamics may have already exerted its effects on the developing myocardium and caused irreversible downstream changes to the ventricle, such a mechanism has yet to be fully elucidated and robustly supported as an explanation for the failure of certain patients to respond to in utero balloon aortic valvulotomy.

Therefore, the data supporting the hemodynamic (or flow) theory, while offering a compelling explanation for progression of certain subtypes of HLHS, remains incomplete. The late features of MS/AA and MS/AS subtypes of HLHS act differently from their LAL model counterparts, and the other HLHS subtypes that arise earlier in development are more likely to arise from genetic or developmental factors outside hemodynamics, since the LAL exerts its effect later in development. Therefore, the most critical piece of data that can be drawn from the hemodynamic theory is that this environmental cue likely plays a large role late in the development of HLHS to either

- initiate the pathogenesis of certain subtypes of HLHS, or
- serve as an essential cue for the disease process to progress.

This recognition provides future researchers with a useful tool to utilize in experimentation, but ultimately, a more foundational variable or a combination of variables need to be identified.

## 4. Primary Myocardial/Endocardial Defect Theory

The ***Primary Myocardial/Endocardial Defect*** model posits that the constellation of features seen in HLHS arises from **intrinsic abnormalities in the formation and maturation of myocardial and endocardial cells**. This theory accounts for the many histological changes seen in HLHS patients and posits a discrete initiating event for HLHS. However, the data used to support this understanding are variable and were obtained under conditions that do not accurately reflect the dynamic environment in which the heart develops.

### 4.1. Histologic and Molecular Evidence

Evidence from both Carrier and colleagues and Liu and colleagues identified patterns of genetic inheritance involving multiple cardiac transcription factors among patients with cardiomyopathies related to HLHS and HLHS itself, suggesting an intrinsic developmental basis of disease [88,93]. These genes are critical in the proliferation of cardiomyocytes and the differentiation of cardiomyocytes, and the assembly of sarcomeres—processes necessary for normal ventricular growth. Identifying these de novo mutations in patients and reproducing the spontaneous emergence of these features in a genetically engineered model lends credibility to the idea that left heart underdevelopment can result from primary myocardial defects, independent of flow disturbances [88,93]. A review from Kritzmire and colleagues provides clinical context, noting that HLHS often presents with a spectrum of structural and functional abnormalities that cannot be explained by altered hemodynamics alone [94]. As mentioned in the previous section, some of the subtypes of HLHS, including MS/AA and MS/AS, develop with minimal early flow disturbance [85,86]. Such evidence suggests that for these subtypes of HLHS, altered hemodynamics may not be the sole initiating factor of disease; and therefore, intrinsic defects in the myocardium/endocardium must be considered as the source of pathogenesis for these HLHS subtypes. However, it is important to consider that cardiac imaging is not currently available at the early stages of development, such as during week 5 and week 6. It is therefore possible that changes to hemodynamics are present and are simply clinically undetectable in the current era. The LAL experiments that best model these forms of HLHS are typically done in a timeframe that mirrors later stages of human development. Because these LAL data do not perfectly model human HLHS, the possibility exists that early flow disturbances are present and may act in combination with other forces, such as intrinsic myocardial/endocardial defects, to predispose patients to these late forms of HLHS.

**Figure 6:**
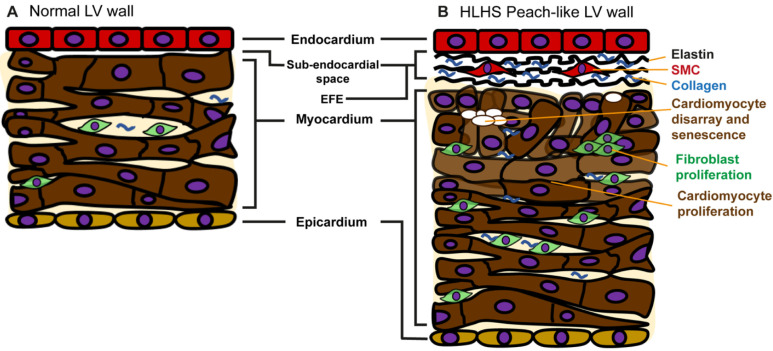
Conceptual illustration comparing normal myocardial architecture (**A**) with that of hypoplastic left heart syndrome (HLHS, (**B**)). The HLHS myocardium is characterized by reduced compaction, aberrant fibroblast content, and disorganized cardiomyocyte layers, suggesting intrinsic developmental immaturity and altered cellular signaling. Reproduced from [87]. Licensed under CC BY 4.0.

One of the most compelling lines of evidence supporting the primary myocardial/endocardial defect theory is the uniformity of histopathological abnormalities observed in hearts with the MS/AA and MS/AS subtypes of HLHS, particularly *endocardial fibroelastosis* (EFE) and myocardial ‘disarray’ [95]. Although the etiology of EFE remains unclear, EFE is widely believed to arise from intrinsic *endocardial* abnormalities. The increased deposition of cells is thought to reflect an elevated number of fibroblasts, which frequently replace or reduce the proportion of cardiomyocytes. This imbalance between fibroblasts and cardiomyocytes may directly contribute to the underdevelopment of the left ventricle, ascending aorta, and aortic arch seen in HLHS [95,96]. Furthermore, subendocardial fibrosis (Figure 6) can encapsulate and mechanically constrain the myocardium, further impairing its growth and reinforcing the hypoplastic phenotype.

In addition to evidence related to histologic changes, compelling evidence from single-cell RNA sequencing analyses provides reasonable mechanistic explanations for the initiation of HLHS. In a study by Miao and colleagues, gene expression of endocardial cells from patients with HLHS, derived from induced pluripotent stem cell (iPSC) lines, showed a significant reduction in several key cardiomyocyte guidance and adhesion proteins that regulate development: NPR3, CDH11, HAPLN1, and ADGRG6 [97]. In addition, expression of alpha-smooth muscle actin (α-SMA) and genes associated with endothelial-to-mesenchymal transition (EndoMT) were also decreased. Such changes have been shown to alter normal EndoMT, NOTCH signaling, and subsequent development of functional myocardium [98,99]. Put together, these aberrant processes cause defective cardiac morphogenesis and subsequent loss of cardiomyocyte number and function. Such defects could be the initiatory event in hypoplasia of valvar and left sided structures.

The reports exploring EFE and dysregulated gene expression in patients with HLHS offer primary explanations for myocardial disease initiation, which are independent of hemodynamic or distinct genetic drivers, and may therefore represent an independent etiology rather than a consequence of some upstream driver of disease.

### 4.2. Primary Myocardial/Endocardial Defect—Theory Limitations (In Vitro Limits and Variable Expression)

One of the central challenges to this theory is that not all patients with HLHS exhibit consistent genetic or cellular markers. Although studies have identified mutations in genes such as MYH6, MYBPC3, and various transcriptional regulators, these findings are often incompletely penetrant and show high phenotypic variability [100,101]. In some of the families in these studies, siblings with identical mutations display markedly different phenotypic cardiac outcomes—or none at all—suggesting that intrinsic myocardial or endocardial abnormalities alone are unlikely to serve as a universal initiating mechanism. This incomplete penetrance and variable expressivity should not diminish the likelihood that such “predisposing” gene mutations/genetic pathways are pathogenic. In other words, such variability does not necessarily rule out these genetic pathways as a major component of the initiation and progression of the various subtypes of HLHS. Instead, this genetic variability reflects the complexity of the genetic landscape of HLHS and supports the fact that modifying factors, such as epigenetic responses, environmental cues, or even in utero exposures to teratogens, may modify certain genetic predispositions or even induce changes in the genetic landscape themselves.

Moreover, although in vitro models using patient-derived iPSCs have revealed altered gene expression with links to impaired endocardial function, these systems lack the biomechanical complexity of the in utero environment. Without exposure to blood flow, pressure gradients, or spatial tissue interactions, these models may capture cell-intrinsic vulnerabilities but fail to explain how such defects modulate malformation of the developing heart [97]. As such, these in vitro models may describe possible susceptibilities, but not necessarily *determinants*, of HLHS. This point is further demonstrated by emerging evidence on the pathogenesis of EFE. Anderson and colleagues, in a commentary on origins of EFE in HLHS, argue that EFE results from altered hemodynamics, rather than as an intrinsic defect. They substantiate this claim by pointing out that patients with HLHS with the mitral stenosis subtype develop EFE at greater rates than patients with HLHS with the mitral atresia subtype [102]. The primary defect theory also struggles to account for flow-responsive clinical outcomes, such as those observed after fetal cardiac intervention. In select cases, in utero balloon aortic valvotomy of the aortic valve has led to preserved or improved growth of left heart structures, allowing for postnatal biventricular circulation [83,84,103]. If HLHS were driven solely by irreversible genetic or cellular defects, such improvement would be unexpected. Put together, the findings of flow-dependent EFE [102] and outcome improvement with in utero balloon aortic valvotomy [83,84,103] suggest that while intrinsic myocardial abnormalities may create susceptibility, hemodynamic forces play a decisive role in modulating the trajectory of disease.

Finally, attempts to model HLHS through genetic or myocardial-specific disruptions in animals have thus far failed to fully recapitulate the human phenotype. Many knockout or transgenic models exhibit early embryonic lethality or unrelated cardiac phenotypes, pointing to a lack of specificity and reproducibility in simulating HLHS via myocardial-only mechanisms [93]. In contrast, surgically induced hemodynamic models (e.g., left atrial ligation) more reliably produce HLHS-like features, underscoring the importance of mechanical factors in shaping left heart development. Importantly, surgically induced hemodynamic models help to explain a subset of HLHS, but clearly not all forms of HLHS. Furthermore, one cannot conclude from these data and these studies that genetic influences do not play a major role in the initiation of HLHS; however, it seems that genetic influences are not likely to be the sole casual event or driver of disease progression, especially in subtypes better explained by disruptions in hemodynamics.

## 5. Valvar Malformation as an Initiating Event in HLHS Theory

According to the ***valvar malformation theory to explain the development of HLHS***, HLHS develops due to congenital stenosis or atresia of the mitral and/or aortic valves. This altered growth of the valves restricts antegrade flow through the left ventricle during a critical window of cardiac development, triggering secondary underdevelopment of the ventricular myocardium and ascending aorta.

This hypothesis is supported by anatomical studies demonstrating a strong correlation between valve-level obstruction and HLHS morphology. In a postmortem analysis of 61 congenital heart specimens, Tláskal and colleagues found that 65% of hearts with HLHS involved significant structural lesions of the mitral and aortic valves. Specifically, 36.1% of specimens exhibited mitral stenosis with aortic atresia, while 32.8% had combined mitral and aortic stenosis—suggesting a consistent pattern of proximal obstruction that aligns with the clinical HLHS phenotype [104]. Furthermore. the co-incidence of bicuspid aortic valve (BAV) and other left-sided cardiac lesions in families with HLHS is supportive of the idea that valvar defects may play an important role in the development of HLHS.

### 5.1. Structural Logic and Hemodynamic Consequences

A central strength of the valvar malformation theory lies in its ability to reconcile both anatomical patterns and flow-mediated developmental responses seen in patients with HLHS. A study by Eckersley and colleagues provides key physiologic support for this hypothesis. In fetuses with HLHS, they observed that overall cardiac output was comparable to controls, but significant disruptions in systemic and cerebral perfusion emerged during the perinatal period [105]. These findings suggest that the issue is not the amount of blood being pumped, but rather where it is being directed—a pattern that aligns with the idea that structural lesions, such as valvar malformations, misroute flow away from the left heart and systemic circulation. This altered distribution of the flow of blood has major developmental consequences. In flow-sensitive models of cardiac growth, it is regional perfusion—not global output—that determines the fate of developing tissues. Boselli and colleagues have shown that shear stress, pressure, and volume loading directly influence endocardial cushion formation, myocardial proliferation, and arterial remodeling—key processes that are disrupted in fetuses with HLHS [106]. When flow through the left heart is diminished by early valve obstruction, these signals are attenuated, setting off a cascade of hypoplastic remodeling.

Further data suggest that patterns of significant left-sided outflow obstruction may exist before definitive ventricular hypoplasia can be observed in fetuses. In a study by Kaltman and colleagues, both retrograde aortic arch flow and lower than normal cerebrovascular resistance were reported in fetuses with HLHS. These findings are consistent with the idea that early valvar malformations restrict normal antegrade aortic blood flow, initiating a cascade of reduced flow and progressive underdevelopment, or hypoplasia, of left-sided cardiac structures and downstream systemic pathways [107]. These findings also provide a clear pathogenic mechanism for the development of HLHS. First, congenital valvar defects cause blood flow to be diverted from the growing left ventricle. Next, this lack of trophic stimuli deprives the growing left ventricle of important mechanical cues that would otherwise signal appropriate left ventricular growth. Finally, this lack of left ventricular growth during key developmental windows leads to the characteristic left-sided hypoplasia seen in patients with HLHS. As previously discussed in this review, one key challenge to this theoretical construct is that in the case of late occlusion of mitral inflow, such as that observed in LAL models, experimental data and HLHS in humans diverge in the demonstrated growth response of the myocardium. In the late types of HLHS in humans, especially in the MS/AA and MS/AS subtypes, thickening of the left ventricular myocardium is present, indicating increased myocardial growth rather than decreased myocardial growth. A primary valvar explanation for the etiology of a subtype of HLHS may fit best with the MA/AA “slit-like” LV phenotype, rather than the MS/AS or MS/AA subtypes. Data from multiple studies in mice suggest that restriction of mitral inflow earlier in gestation (i.e., day E.14–16 correlating to around week 8 and 9 in humans) produces a non-apex-forming slit-like ventricle and retrograde aortic flow, similar to the human MA/AA subtype of HLHS [81,108]. These data suggest that an *early* insult to mitral inflow, such as a congenital atresia of the mitral valve, may be a strong factor in the etiology of this particular MA/AA subtype of HLHS.

### 5.2. Valvar Malformation as an Initiating Event in HLHS—Theory Limitations (Anatomic Exceptions and Mechanistic Ambiguity)

A potential limitation of the valvar malformation theory is that this theory fails to account for the origin of the valvar malformations themselves during development; of course, limited information exists altogether about what causes valvar defects (genetic or otherwise) in humans, so it is not surprising that this information is not available for patients with HLHS. While mitral or aortic stenosis is often present in HLHS, the valvar model does not offer a mechanistic explanation for why these malformations occur. In this way, it risks describing the first observable consequence, rather than the true initiating cause. This important limitation of the valvar malformation theory therefore supports a genetic mechanism in which the primary defect is valvar.

In addition, the heterogeneity of anatomy in patients with HLHS cannot be fully explained by the valvar malformation theory. A large study conducted by Crucean and colleagues categorized subsets of the phenotypes of HLHS. Of the three main HLHS phenotypes described by this study, one subgroup showed normal valvar anatomy and valvar function but maintained hypoplastic left ventricular anatomy [109]. This piece of data would suggest that a significant number of cases of HLHS do not require the abnormal function of valves to be present; and therefore, these data undermine the idea that valvar defects are the common initiating event and instead point to a broader spectrum of initiating mechanisms. This limitation of the valvar malformation theory would seem to support a primary defect in ventricular development as the cause at least for a subset of patients with HLHS. Clearly, different subtypes of HLHS exist, some of which might arise from a primary valve defect, whilst others do not.

Furthermore, the temporal relationship between valvar obstruction and left heart hypoplasia is difficult to resolve. It remains unclear whether restricted flow due to valvar stenosis causes ventricular underdevelopment, or whether the ventricle is intrinsically hypoplastic and unable to support normal valvar development. Taken together, the most likely scenario is that there must be a combination of “hits” impacting valvar and ventricular development. The differences may lie in which is the primary versus the secondary event, developmentally. Without early developmental imaging or molecular markers, distinguishing cause from effect remains speculative.

Finally, as discussed previously in this review, the response of fetuses and neonates to fetal valvuloplasty is variable, and this variability presents a major challenge to the valve-first model. Although in utero balloon aortic valvotomy has been shown to improve outcomes in some patients with HLHS, many still go on to develop HLHS [83,84,103]. Like the subset of phenotypic cases that have hypoplastic left ventricles despite normal valvar anatomy, this subset of patients who fail to improve despite intervention provides evidence that would suggest valvar malformation is likely not the primary insult that drives progression of disease in certain subtypes of HLHS. Therefore, while valvar malformation likely plays a significant role in the development of some subtypes of HLHS and guides the most effective in utero clinical intervention for select patients, the valve-first theory ultimately remains unlikely as the etiology of many cases of HLHS.

## 6. Genetic and Epigenetic Factors Theory

The ***genetic framework for understanding the etiology of HLHS*** proposes that inherited or de novo mutations, as well as epigenetic modifications, disrupt key cell signaling pathways in the embryonic heart. Such disruptions can cause dysregulation of normal morphogenesis and result in the observed pathologic anatomy and function of hearts with HLHS. Current data demonstrates that HLHS can be heritable; however, no research has conclusively provided a clear, reproducible genetic model for the disease.

### 6.1. Genetic Risk and Developmental Pathways

**Figure 7:**
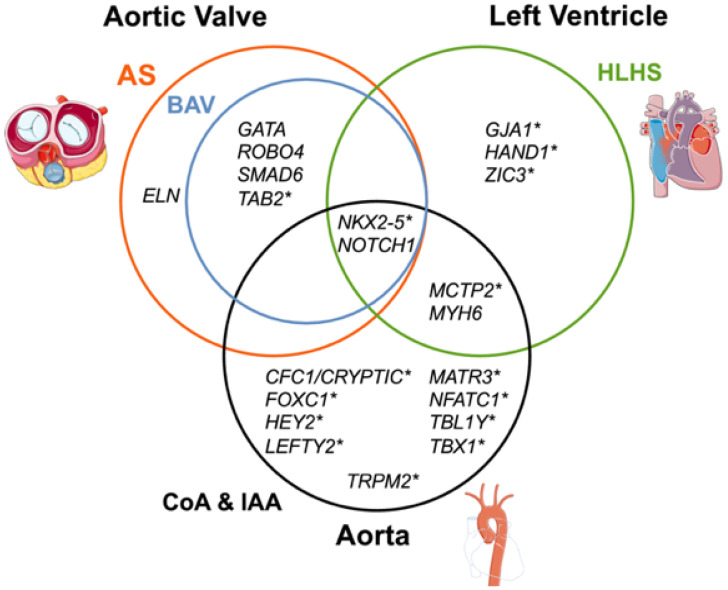
Overlap of genes implicated in HLHS, aortic valve disease, and coarctation of the aorta. Genes such as NOTCH1, HAND1, and MYH6 appear centrally, reflecting shared developmental pathways across left-sided obstructive lesions. Reproduced from [118]. Licensed under CC BY 4.0.

Following the identification of HLHS heritability, a growing number of genetic signaling pathways have been associated with HLHS. One of the most studied is the NOTCH1 signaling pathway, which is a signal cascade that plays an essential role in cardiac morphogenesis and especially in the differentiation of progenitor cells into distinct cardiac cell types. In iPSC models with disrupted NOTCH1 signaling, taken from human patients with heterozygous NOTCH1 mutations, cardiac myocytes became underdeveloped and disorganized [110,111]. This finding alone implies that the NOTCH1 pathway may play a key role in the development of HLHS, but other candidate genes with suspected or demonstrated involvement in HLHS have been identified, including HAND1 [112], GJA1 [113], NKX2-5 [114], FOXC2 and FOXL1 [115], MYH6 [116], TBX5 [117], and the gene cluster SAP130/PCDHA9 [93]. Although many of the findings remain preliminary, the dysregulation of these genes consistently impacts normal cardiac development and remains a useful framework to uncover the origin of HLHS (Figure 7).

Moreover, many patients with HLHS demonstrate extracardiac anomalies. Current data suggests that up to 28% of patients with HLHS have coexisting genetic syndromes or major non-cardiac malformations, including diaphragmatic hernia and omphalocele [119]. These multisystem abnormalities suggest a common underlying disruption of early embryonic gene networks, reinforcing the idea that HLHS can arise as part of a broader developmental disorder with an initiating genetic insult. It is important to emphasize that the subtypes of HLHS that seem to develop early in gestation, that is with features of HLHS detectable on early fetal screenings, are likely influenced more or even solely by these genetic risks and early developmental pathways. Meanwhile other subtypes of HLHS that develop later in gestation are likely more heavily influenced by environmental factors such as flow defects.

### 6.2. Genetic and Epigenetic Factors—Theory Limitations (Architectural Complexity and Predictive Uncertainty)

Some studies have been able to reproduce certain phenotypes of HLHS in mice; however, the data is inconsistent and nonrepresentative of the entire spectrum of pathology seen in HLHS. For example, in the pivotal murine study conducted by Liu and colleagues, briefly discussed in previous sections of this review, certain HLHS phenotypes emerged from a combination of several mutations rather than a single shared variant, and the combinations themselves were incompletely penetrant. Although the evidence for the requirement for inheriting multiple genes (beyond SAP130/PCDHA9) is circumstantial, this finding would suggest that HLHS arises from genetic disruptions across interconnected developmental pathways, and that if such combinations of genetic disruptions do exist, they remain beyond our understanding at this time [93].

Epigenetic mechanisms may further complicate this problem [120,121]. Histone modifications, DNA methylation, and non-coding RNAs have been proposed as regulatory forces that influence cardiac development. Although these epigenetic factors remain more poorly characterized than the genetic pathways, these epigenetic factors may still contribute to the striking variability in the clinical presentation of HLHS and may interact with undescribed environmental influences to produce the heterogenic phenotypes seen in patients with HLHS [120,121].

Based on the current literature, genetics play a definitive role in the development of HLHS, but that exact role remains unknown. Although future research may provide useful information on the extent that genetics influence disease progression or which genetic networks may have a role in disease initiation, the genetics of HLHS are extremely complicated. Therefore, the early forms of HLHS that may be the most influenced by genetic forces will be difficult to model effectively through a purely genetic framework. Future research may be better served by identifying a more reproducible factor related to the initiation and progression of disease in these early types of HLHS. In doing so, our understanding of how gene networks and epigenetic modifications converge to exert their effects in the early forms of HLHS may become more comprehensive and robust.

## 7. Multifactorial Theory

The ***multifactorial theory to explain the development of HLHS*** proposes that HLHS arises from the interplay of partially penetrant genetic variants, altered hemodynamics, disrupted molecular signaling pathways, and environmental exposures. This understanding of the etiology of HLHS aims to accomplish the following objectives:

- explain the phenotypic heterogeneity,
- reconcile the positive explanatory power of disparate theories,
- identify the developmental windows for possible interventions, and
- stratify patients according to which pathogenic factor is the dominant driver of disease progression.

### 7.1. Synthesis Across Mechanisms

The major strength of the multifactorial theory lies in its ability to resolve the central limitation shared by the other models discussed thus far: *each theoretical model can explain a subset of the features of HLHS, but none account for the full phenotypic spectrum*. For example, while studies of fetal intervention demonstrate that restoring antegrade flow through the aortic valve can improve outcomes in select cases, not all fetuses respond equally to in utero balloon aortic valvotomy, suggesting that genetic susceptibility or intrinsic myocardial immaturity may constrain the capacity for catch-up growth [122]. Genetic data further support a multigenic origin. Studies have shown that no single gene mutation accounts for most cases of HLHS, but rather that interacting variants in pathways like NOTCH, FGF, and BMP signaling contribute to risk in aggregate [123,124]. Parker and Landstrom argue that HLHS should be understood as part of a continuum of left-sided obstructive lesions, with overlapping molecular contributors that vary in expression across individuals [118]. This concept explains in part why different patients—even with similar anatomy—can follow markedly different clinical trajectories.

At the signaling level, recent molecular analyses have revealed disruptions in cardiac transcription factors, extracellular matrix regulators, and mechanosensitive genes, suggesting that a combination of impaired communication between genetic programs and mechanical forces may lie at the heart of the pathogenesis of HLHS [120].

Environmental contributors also likely play a role in the pathogenesis of HLHS. In a study by Simeone and colleagues, up to 20% of patients with HLHS were exposed to known environmental risk factors, including maternal diabetes, obesity, and teratogenic exposures. These data suggest that environmental factors may influence the development of HLHS and contribute to the susceptibility of patients to the disease [125].

The strength of the multifactorial framework for understanding HLHS is that it offers a flexible, comprehensive hypothesis that more accurately reflects the dynamic environment in which congenital heart disease develops. It can account for influences outside of the purely genetic or purely hemodynamic and allow for research to explore factors previously uninvestigated in the literature.

### 7.2. Multifactorial Theory—Theory Limitations (Broad Scope, Narrow Precision)

The primary limitation of the multifactorial model is its lack of specific, definable mechanistic interactions. Although it accommodates the observed complexity of HLHS, this framework is difficult for testable hypotheses to develop within. Because so many different combinations of factors could theoretically contribute to the development of HLHS, the model risks becoming descriptive rather than mechanistic.

This problem is most evident in the research that has been done to solve the etiologic puzzle. Most current studies analyze forces associated with disease, such as flow disturbances or genetic abnormalities, in isolation. As a result, integrated datasets that could more clearly define the relationship of molecular and imaging data in the same patient with HLHS remain scarce. Without this kind of systems-level integration to facilitate defining how specific combinations of risk factors interact to cause HLHS, one of the primary problems the multifactorial theory tries to resolve will remain unsolvable.

Thus, the multifactorial theory is a good account of the driving forces behind HLHS, but it is one of the least useful for researchers to test and for clinicians to utilize at the bedside. Nevertheless, this *multifactorial theory to explain the development of HLHS* may very well approximate a reality where many different combinations of factors could theoretically contribute to the development of HLHS.

## 8. Future Directions

In order to move beyond associations and toward a causal explanation of the etiology of HLHS, a bold and necessary new approach is needed. Namely, the identification of specific cardiac progenitor cell populations onto which genetic, hemodynamic, and environmental forces converge to exert their influence on initiation and progression of disease. This understanding is analogous to the results of a study conducted by Nie and Bronner and published in 2015 [126]. Nie and Bronner observed that knockdown of an HLHS candidate gene, ETS1, in specific cell lineages, namely the cardiac neural crest and the cardiac mesoderm, led to distinct pathologic anatomies in frog hearts. When ETS1 was knocked-down in the cardiac neural crest lineage, aortic arch formation was defective; whereas, when ETS1 was knocked down in the cardiac mesoderm, the resulting pathology was localized to the endocardium and the outflow tracts [126]. These lineage-specific outcomes reinforce the idea that genetic perturbations operate within discrete progenitor populations to create distinct structural pathologies.

A similar idea was explored in a paper by Ahola and colleagues. In a retrospective study of patients at their institution with HLHS, the authors noted a 117-fold increase in the incidence of Hirschsprung’s Disease among the population of patients with HLHS compared to the general population [127]. Such an increase in incidence would suggest that neural crest cell lineages play a role in these patients with the combination of HLHS+ Hirschsprung’s and support the idea that subtypes of HLHS may be understood through the dysregulation of cell populations and their offspring.

This concept opens many possibilities to a new understanding of the etiologies of the various subtypes of HLHS. ***Specific cell lines, disturbed by alterations in genetic regulations or other environmental influences, could serve as the initiating events at the foundation of each observed subtype of HLHS***. For example, the disturbance of cells responsible for normal valvar development may serve as the “ligation event” that causes downstream left ventricular hypoplasia observed in LAL studies. Similarly, specific endocardial cell line disruptions may be responsible for EFE and the resultant left ventricular hypertrophic subtype of HLHS. Together, these insights point toward a cellular-lineage model of initiation of HLHS. That is, identifying defective cell lineages is the foundation for understanding the etiology of the various subtypes of HLHS. This thesis is not to say the entire spectrum of HLHS shares a common etiology. Rather, it emphasizes that understanding how each subtype of HLHS develops starts at the cellular level.

A 2017 study conducted by Crucean and colleagues, previously cited in this review, approached understanding HLHS in such a way. Crucean and colleagues analyzed hearts with HLHS and categorized HLHS into three distinct subtypes based on left ventricular morphology [109]: (1) slit-like, (2) miniaturized, and (3) thickened with EFE. Using lineage tracing with Cre technology (e.g., Nkx2–5-Cre, Mef2c-AHF-Cre and Wnt1-Cre), the authors demonstrated that several sub-populations of cardiac progenitor cells contribute significantly to distinct anatomic structures involved in HLHS. From these findings, the authors concluded that HLHS should be stratified by ventricular morphology, with each form reflecting perturbation of a unique progenitor lineage [109].

These emerging data suggest that HLHS can be understood in a completely different way than previously outlined in the literature. The cell subpopulation basis of disease is a uniquely dynamic framework within which to conduct research that has the potential to unify the associated strengths of the various hypotheses outlined previously in this review. The cellular understanding also has the distinct benefit of potentially explaining each subtype of HLHS individually instead of attempting to resolve the initiation and progression of each subtype of HLHS within one contributing factor, such as genetic perturbation or hemodynamic signaling. In this way, the cellular understanding of HLHS provides a unified framework for experimentation while allowing for the diversity of phenotypes seen across the spectrum of HLHS.

Moreover, this hypothesis can rectify the main downside of the popular and comprehensive multifactorial understanding. That is, cell populations are distinct, testable variables that are highly vulnerable to disruptions in the myriads of forces at play in normal development. This concept would account for the apparent multi-variable contributions to the initiation and progression of HLHS while being grounded in experimentally testable, describable explanations.

The first challenge in testing such a hypothesis is to identify the progenitor cell populations that give rise to the structures that are consistently malformed in patients with HLHS: the left ventricle, aortic and mitral valves, ascending aorta, and aortic arch. This population of progenitor likely includes

- Contributions from FHF progenitors (especially for left ventricular myocardium),
- SHF cells (which contribute to outflow tract and valvar structures)
- Endocardial precursors, particularly those undergoing EndoMT, and
- Cardiac Neural Crest Cells.

To directly evaluate this hypothesis, future experimental strategies could focus on resolving temporally and transcriptionally distinct subpopulations within the FHF, SHF, Cardiac Neural Crest, and endocardium during normal cardiac development and determining how their perturbation could generate the divergent morphologies of HLHS. The first step could be the utilization of single-cell RNA sequencing (scRNA-seq) transcriptomics of murine fetal hearts to define the transcriptional identity of primitive endocardial or FHF/SHF cell niches. Once these niches have been identified, spatial transcriptomic technology can be employed to link transcriptionally distinct populations to certain anatomic locations of the developing heart, such as the inflow tract or the left ventricle.

Once temporally, spatially, and transcriptionally distinct candidate progenitor populations are identified, their functional role in left heart morphogenesis must be validated. This validation can be accomplished through the following approaches, although this list is not exhaustive:

- Genetic perturbation using CRISPR/Cas9 knockout or knockdown of lineage-specific transcription factors;
- Cell–cell competition experimentation to examine how adapted HLHS-type progenitor cells are to colonize certain developmental niches
- In vitro organoid or cardiac microtissue models, in which niche-specific endocardial cells are subjected to hemodynamic loading and extracellular matrix interactions.

This proposed approach is ambitious in scope and technically demanding, but the potential payoff is significant. Identifying a root progenitor population for each subtype of HLHS would transform HLHS from a syndromic cluster of anomalies into a definable developmental failure—opening multiple new avenues, including avenues for

- Molecular diagnosis,
- Risk stratification, and
- Potentially regenerative therapies targeted at rescuing or replacing compromised progenitor pools in utero.

## 9. Limitations

Data from a morphologic study conducted by Stephens and colleagues suggest that HLHS arises as an acquired defect during fetal life. The authors noted extraordinary variability across 119 specimens from patients with HLHS, and they note that such heterogeneity must arise after interventricular septation, rather than from perturbations in embryogenesis [128]. This suggestion is made more plausible by the recent data from Rahman and colleagues, discussed in an earlier section of this review, which showed that surgical embolization of the LA in mice can reproduce HLHS phenotypes with full penetrance, a model that genetic studies have failed to provide researchers [81]. Taken together, the data from these studies pose a similar challenge to the cellular understanding as an etiologic explanation of HLHS as many of the other theories outlined in this review. That is, cells may explain only select subtypes of HLHS rather than serve as a basis for understanding each distinctly. Data suggests some subtypes of HLHS develop later in fetal life, after potential candidate cell subpopulations have matured and differentiated; and therefore, the cellular based hypothesis may also fail to account for these cases of HLHS. Therefore, while this review emphasizes the potential unifying power of cell progenitor populations to explain the etiology of each subtype of HLHS, the results of Stephens and colleagues [128] and Rahman and colleagues [81] underscore that this future direction remains a hypothesis driven framework to guide experimentation, rather than a settled determination.

Another key limitation to this understanding lies in the ability to model HLHS effectively. Currently, no animal model has consistently reproduced the entire phenotypic spectrum of human HLHS; and the models that do exist, such as LAL, remain imperfect. As a result, the task of identifying key cell populations in an embryonic tissue (such as the FHF or SHF) that may reliably reproduce HLHS features is a daunting and arduous task. It is important to emphasize, therefore, that a reliable model for each HLHS subtype is of paramount importance to future investigation into potential cellular etiologies.

## 10. Conclusions

The title of our manuscript asks the following question: “***Is There a Unified Etiology of Hypoplastic Left Heart Syndrome?***” Based on the arguments made throughout this manuscript that evaluate the various the genetic, structural, and hemodynamic models of initiation of disease, we believe that ***the significant phenotypic variability across the spectrum of HLHS (i.e., the different anatomic subtypes for “classic” HLHS) most likely reflects different underlying etiologies and mechanisms***. At the very least, it is very likely that the ***timing*** of the insult is critical in determining anatomic subtype. As described in our manuscript, different mechanisms likely differ in terms of timing in development. For example, the flow theory is a late event (as shown in models with the chick embryo and the mouse), whereas the genetic etiologies likely occur early in cardiac development. In the final analysis, based on the published data and the arguments within this manuscript, it seems naive to think that there is a single unifying mechanism explain all forms of HLHS.

Table 1 provides a sequential summary of the proposed etiologies for hypoplastic left heart syndrome (HLHS) discussed in this manuscript. Despite decades of research, the precise etiology of HLHS remains unresolved. Although current theories (Table 1) can account for aspects of the disease, none of these theories explain the full picture of human HLHS. Key questions remain unanswered, including:

- The temporal sequence of defects,
- The origin of structural underdevelopment, and
- The source of anatomic heterogenicity.

This paper has outlined a multitude of individual potential etiologies of HLHS (e.g., hemodynamic signaling and genetic predispositions) that have been shown to contribute in some way to produce the phenotypic spectrum of HLHS. Current data suggests that each subtype of HLHS has a unique etiology and depends on different developmental forces to progress into the disease known to clinicians. Understanding the importance of this concept, we propose that the unique etiologies of each subtype of HLHS may share a common thread: that developmentally traceable and transcriptionally distinct progenitor pools of cells lie at the foundation of the initiation and progression of disease. This framework shifts focus away from identifying the forces associated with the development of each subtype of HLHS and instead points toward a potential keystone onto which these forces converge. In doing so, this proposed framework may provide a deeper understanding of the pathogenesis of each subtype of HLHS.

The growing recognition of HLHS as a spectrum has led to a more integrated model that incorporates the positive aspects of each theory while leaving room for other components to explain that which each theory cannot individually explain. Such a framework is useful to encompass every aspect of HLHS, but is unable to provide pathways forward for researchers to identify a common, definable link between the various associations. This paper proposes such a pathway forward. Our proposed pathway forward is to focus research on identifying a specific cardiac progenitor cell population whose failure underlies the development of each subtype of HLHS. This approach has the potential to unify existing theories and to clarify the developmental cascade that leads to left-sided hypoplasia.

As researchers begin to unravel a unified etiology for HLHS, their primary target must be as dynamic, heterogeneous, and ultimately reliant on molecular and environmental factors as the syndrome itself. Cell populations would fit such a bill. By combining modern tools in single-cell biology, genetics, biomechanics, and fetal imaging, the field is now poised to identify such pools of cells. In doing so, research may progress from the correlative to the causative, opening doors to treatments beyond the operating room.

## Figures and Tables

**Table 1:** Sequential Summary of Proposed Etiologies for Hypoplastic Left Heart Syndrome (HLHS).

Etiology	Concept Summary	Key Strengths	Key Limitations	Current Standing
**1. Flow Theory**	Abnormal fetal blood flow alters chamber and valve development.	Robust support from animal models (e.g., left atrial ligation); clinical improvement following fetal intervention; explains dynamic remodeling of left heart structures.	Direction of causality remains unclear; not all HLHS cases show early flow disturbances; cannot fully explain endocardial fibroelastosis (EFE) or intrinsic myocardial defects.	**Widely accepted** as a modifier of disease progression, but not sufficient to explain initiation.
**2. Primary Myocardial Defect**	Intrinsic abnormalities in cardiomyocyte or endocardial development lead to impaired growth of the left ventricle and associated structures.	Consistent histopathology (EFE, myocardial disarray); spontaneous HLHS-like features in genetically engineered mice; transcriptomic evidence from patient-derived iPSC models.	Incomplete penetrance of genetic findings; in vitro models lack biomechanical context; fails to explain positive response to restored flow in some cases.	**Emerging evidence** supports causative role in select cases; not universally explanatory.
**3. Valvar Malformation**	Structural malformations of mitral or aortic valves restrict antegrade flow, triggering secondary hypoplasia of downstream structures.	Aligns with fetal imaging and postmortem data; explains regional perfusion changes and outflow abnormalities; integrates well with flow-sensitive developmental responses.	Does not explain origin of valvar defects; some HLHS subtypes lack significant valve obstruction; limited mechanistic insight into underlying cause.	**Contributory**, especially in morphologically severe or early diagnosed cases.
**4. Genetic/Epigenetic Factors**	Inherited or de novo mutations, as well as epigenetic changes, disrupt cardiac development and spatial patterning.	High heritability; links to known cardiac transcription factors (e.g., NOTCH1, HAND1); supported by syndromic associations and animal models; accounts for multi-organ anomalies.	Genetic heterogeneity; variable expressivity; incomplete reproduction of human HLHS in animal models; low predictive utility for individual cases.	**Strongly supported as a core risk factor**, though not always sufficient on its own.
**5. Multifactorial Model**	HLHS arises from a combination of genetic susceptibility, structural defects, altered hemodynamics, and environmental exposures.	Reconciles competing models; accounts for inter-individual variability; supported by evidence of partial responses to interventions and interacting developmental pathways.	Broad and non-specific; difficult to test experimentally; lacks predictive precision or clinical applicability in its current form.	**Most comprehensive theory**; reflects current direction of HLHS research.

## Data Availability

No new data were created or analyzed in this study.
